# Supplementary material for: Oxidation of F-actin controls the terminal steps of cytokinesis
Source: Nat Commun. 2017 Feb 23;8:14528. doi: 10.1038/ncomms14528 (PMC5331220; doi:10.1038/ncomms14528)
Supplement: Supplementary Information — Supplementary Figures, Supplementary Tables and Supplementary References. [file ncomms14528-s1.pdf]

SUPPLEMENTARY INFORMATION

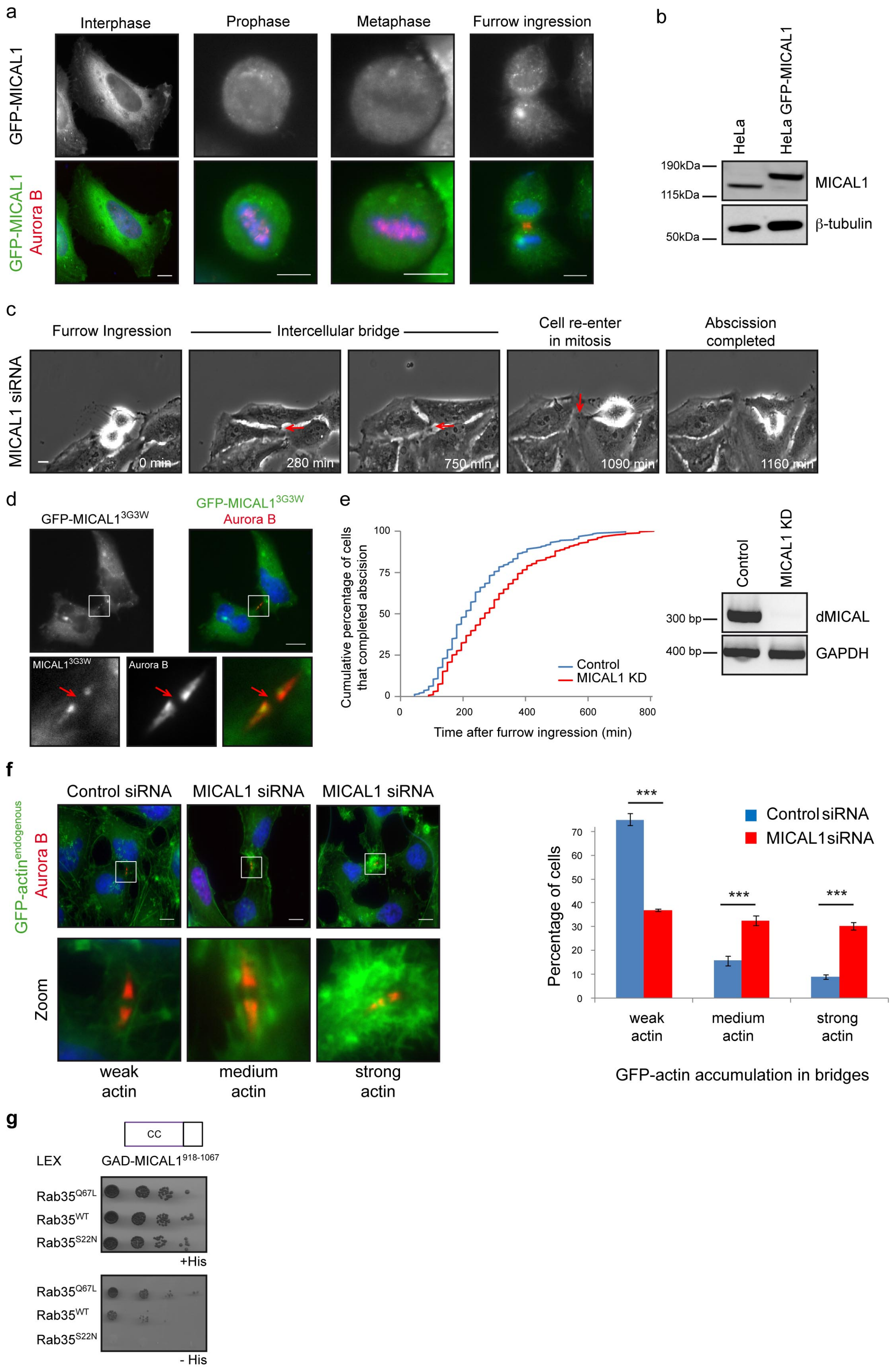

Supplementary Figure 1: Function of MICAL1 and dMICAL in cytokinesis

(a) HeLa transfected with GFP-MICAL1 (green) were stained with Aurora B (red). Scale bars, 10 μm.
(b) Western blot from HeLa (parental cell) and HeLa GFP-MICAL1^{endogenous} cell line extracts. Loading control: β-tubulin.
(c) Snapshots from a time-lapse phase-contrast microscopy movie of MICAL1-depleted cells. Scale bars, 10 μm.
(d) HeLa cells transfected with GFP-MICAL1^{3G3W} catalytically-dead mutant (green) were stained with Aurora B (red). Scale bars, 10 μm.
(e) Distribution of the abscission times in control- and MICAL1-depleted *Drosophila* S2 cells (N= 3). p = 0.000 between red and blue curves (KS test). n= 249-272 cells per condition. RT-PCR for *GAPDH* and *dMical* at Day6.
(f) Left: HeLa cell line expressing actin tagged by GFP following TALEN excision and homologous recombination at the endogenous locus (GFP-actin^{endogenous}) were stained with AuroraB (red) and DAPI (blue) in control- or MICAL1-depleted cells. Right: Quantification of the intensity of the GFP-actin^{endogenous} in bridges after control and MICAL1 depletion (N= 3). Error bars represent standard deviations. ***: p< 0.001 (two-way ANOVA). n= 192-234 cells per condition. Scale bars, 10 μm.
(g) *S. cerevisiae* reporter strain expressing indicated GAD- and LEX fusion proteins, and grown on selective medium with or without Histidine.
In all figures, Red arrow: Flemming body.

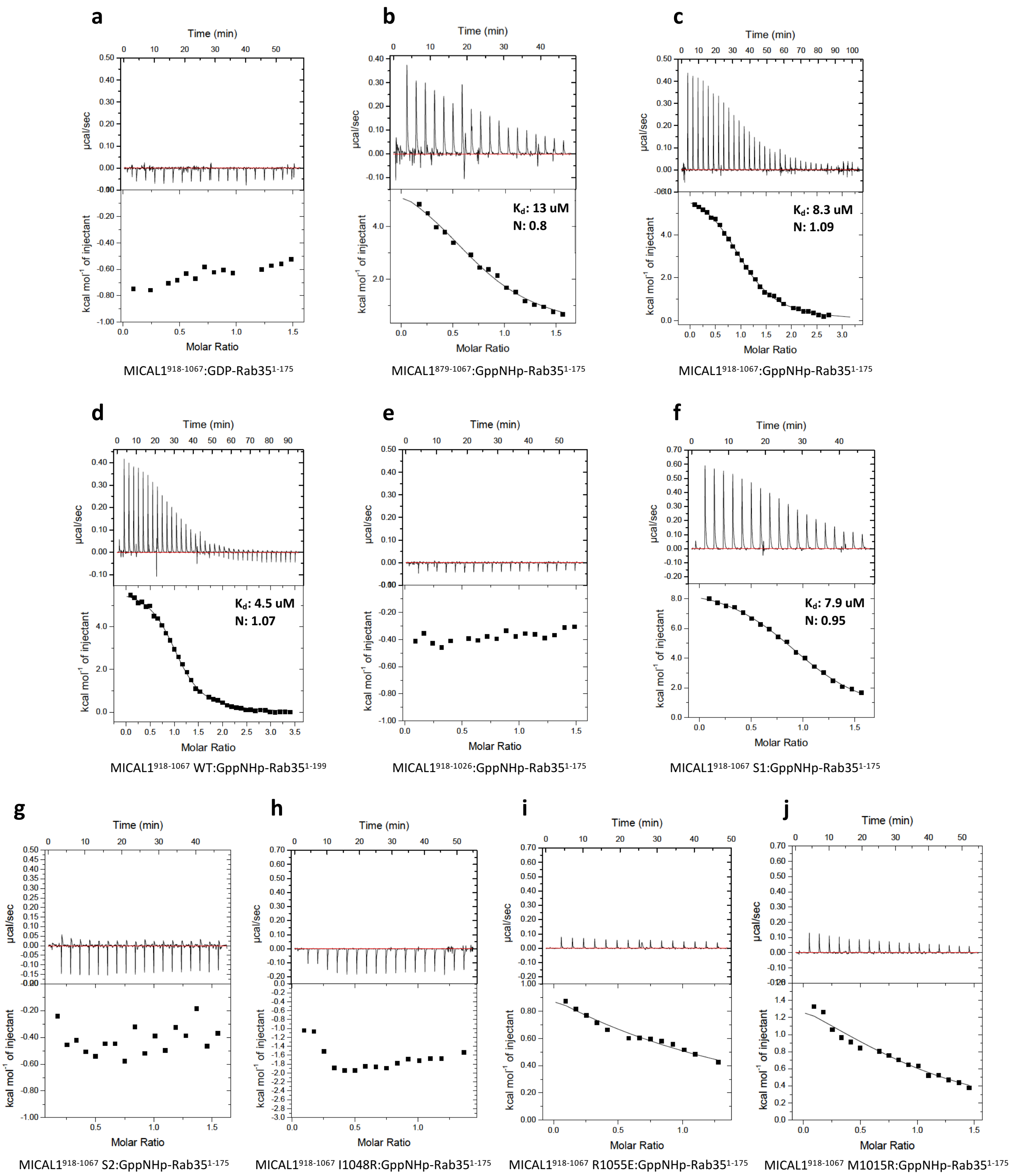

Supplementary Figure 2: Rab35 / C-terminal MICAL1 constructs Interaction by Isothermal Titration Calorimetry (ITC)

The top panels show the baseline corrected titration data, and the bottom panels show the binding isotherm fitted for the interactions between GDP-Rab35$^{1\text{-}175}$ and MICAL1$^{918\text{-}1067}$ wild-type (a) or GppNHp-Rab35$^{1\text{-}175}$ and: MICAL1$^{879\text{-}1067}$ wild-type (b), MICAL1$^{918\text{-}1067}$ wild-type (c), MICAL1$^{918\text{-}1026}$ (e), MICAL1$^{918\text{-}1067}$ S1 mutant (E946K, V950D, E953K, V971E, L975R, V978E) (f), MICAL1$^{918\text{-}1067}$ S2 mutant (R1012E, M1015R , L1034K, V1038E) (g), MICAL1$^{918\text{-}1067}$ I1048R (h), MICAL1$^{918\text{-}1067}$ R1055E (i) or MICAL1$^{918\text{-}1067}$ M1015R (j). Moreover, titration of GppNHp-Rab35$^{1\text{-}199}$ with MICAL1$^{918\text{-}1067}$ wild-type (d) was tested and show that the Rab35 tail doesn't contribute much to the affinity. The results were fitted using one site binding mode resulting in estimation of the complex dissociation constants (K_D) and interaction stoichiometry (N).

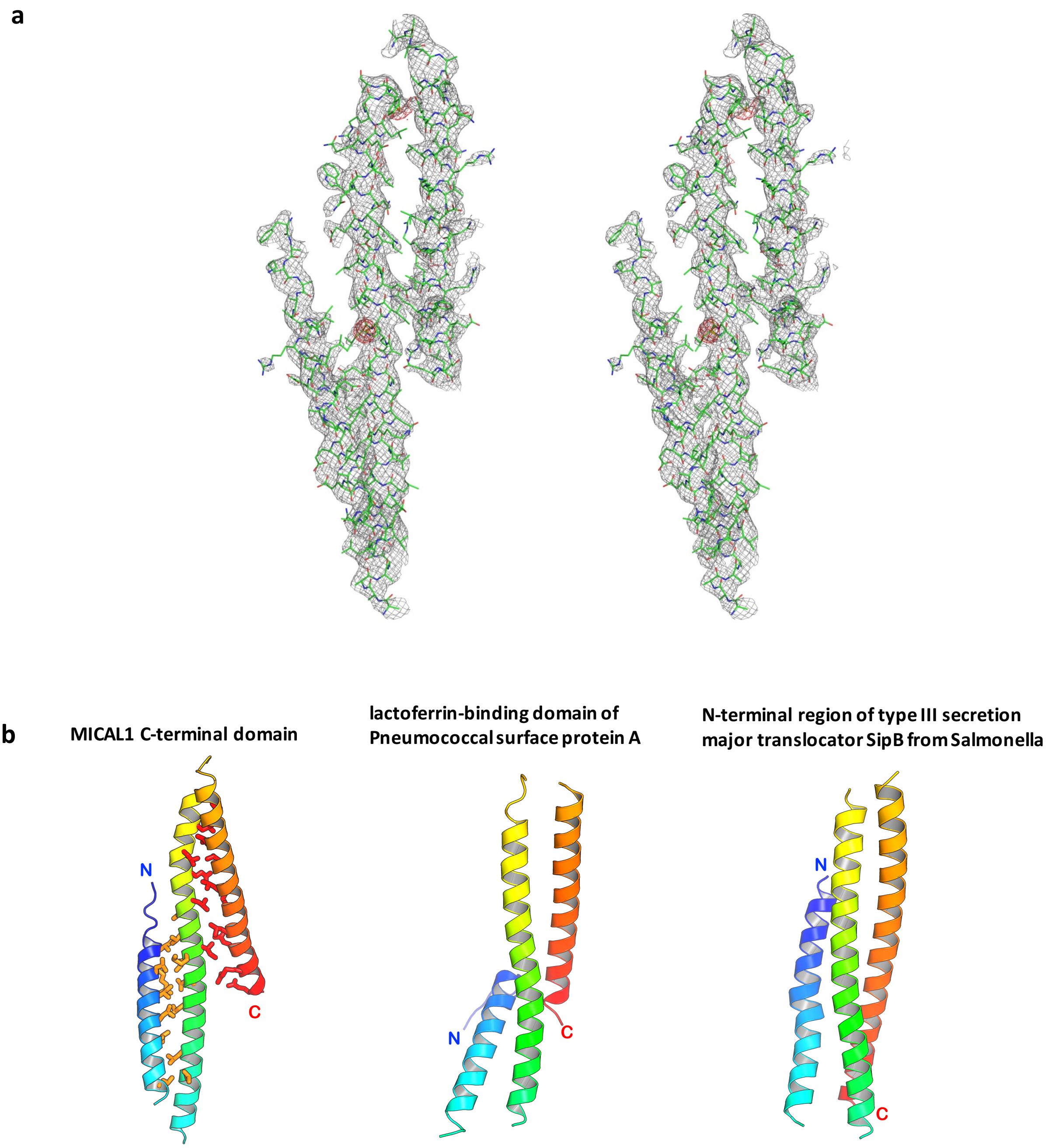

Supplementary Figure 3: MICAL1 C-terminal domain crystal structure

(a) The MICAL1 C-terminal domain structure is shown in stereo view with a sigma-weighted 2Fo-Fc map at 3.3Å resolution, contoured at 1.0 σ (grey) and anomalous difference map contoured at 4σ (red). (b)The structure of MICAL1 corresponds to a sheet of three anti-parallel helices. Note that it is difficult to predict whether helical regions form a single alpha-helix[1] or pack as anti-parallel or parallel helices[2]. This structure provides insights about how alpha-helices that have characteristic coiled-coil repeat patterns can pack. The first (H1) and the last helices (H3) of MICAL1 bind to the helix-2 (H2) through coiled-coil interaction (shown with side chains in sticks). The distribution of hydrophobic residues on the central H2 helix orients the other two helices. Thus, the MICAL1 C-terminal domain assembles in an original, flat, alpha-helical sheet structure, rather than a three-helix bundle. This C-terminal domain is compared with two three-helix fold of closest topology found with the PDBe fold server (https://www.ebi.ac.uk/msd-srv/ssm/cgi-bin/ssmserver): lactoferrin-binding domain of Pneumococcal surface protein A (PDB ID 2PMS)[3], and N-terminal region of type III secretion major translocator SipB from Salmonella (PDB ID 3TUL)[4]. The three protein folds consist of three consecutive helices shown in rainbow colors that interact in an antiparallel manner, but in MICAL1, the H1 and H3 helices do not interact and orient themselves to form a flat curved sheet.

```
                                        -----------H1--------------
                                        a--d---a--d---a--d---a--d
MICAL1_Human      911  RTLLRRAKEEEMKRFCKAQTIQRRLNEIEAALRELEAEGVKLELALRRQ---------------------------SSS--- 962
MICAL1_Mouse      898  RTLMRRAKEEEMKRFCKAQAIQRRLNEIEATMRELEAEGTKLELALRKE---------------------------SSS---
MICAL1_Chicken    992  RTLNRHAREAQMKRFCKAQAIQRRLEEIEVTFRELEQQGIKLEKFLRED---------------------------SDS---
MICAL1_Zebrafish  1046 KTLERKAKMTEIQRFHKAQSIQRRLEEIEVTFKELEEKGVELERALRGE---------------------------TGT---
MICAL_FruitFly    4543 RAISKASRQAELKRLRIAQEIQREQEEIEVQLKDLEARGVLIEKALRGEAQNIENLDA------------------------
MICAL_ApisFlorea  2595 KTTKRIARQAQLKRLRMAQEIQRKLEETEVKQRELESRGVSVEKALRGE---------------------------GE---C
Mical3_Human      1834 KAARRQAKQEELKRLHRAQIIQRQLQQVEERQRRLEERGVAVEKALRGE---------------------------AGM---
MICAL3_Mouse      1825 KAARRQAKQEELKRLHRAQIIQRQLEQVEEKQRQLEERGVAVEKALRGE---------------------------AGM---
MICAL3_Chicken    2018 KAARRQAKQEELKRLHRAQIIQRQLEQVEEKQRQLEERGVAVEKALRGE---------------------------AGM---
MICAL3a_Zebrafish 1488 RAARRQAKQEELRRLHRAQIIQRQLEQVEVKQRQLEEKGVAVEKALRGEADFWEDS-----S-----TSVLLDVHLCGM---
MICAL3b_Zebrafish 1809 KAARRQAKQEELKRLHRAQMIQRQLEQVEEKQRQLEERGVAVEKALRGEADYWGES-----N-----YSEILDLHLGGM---
MICAL3_Lizard     2065 KAARRQAKQEELKRLHRAQIIQRQLEQVEEKQRQLEERGVAVEKALRGEAVEPNAG-----TPRRRPLSFCPCCAHEGM---
MICAL-L1_Human    674  ----------QADQYIPEEDIHGEMDTIERRLDALEHRGVLLEEKLRGGLN-------------------------------
MICAL-L1_Mouse    681  ----------QADQYIPEEDIYGEMDNIERQLDALEHSGVLLEEKLRGGAN-------------------------------
MICAL-L1_Chicken  764  ----------QTDQYIPEEDIYGEMDAIEHQLDQLEHRGVILEEKLRSSEN-------------------------------
MICAL-L_FruitFly  772  ----------KVLQRLPLQEIRHEFEIIAVQQLGLEKQGVILEKMIRDRCERS--LDATDTDGP-----------ESAEVLT
MICAL-L_Lizard    733  ----------QSDQYVPVEDIYGEMDSIEQQLDELEHRGVELERKLRSMENVRERLNCSSLKRWEQDISFISQLHSLGEV--
MICAL-L2_Human    730  ----------LHPDYLSPEEIQRQLQDIERRLDALELRGVELEKRLRAAEG-------------------------------
MICAL-L2_Mouse    836  ----------LHPDYIPQEELQRQLQDIESQLDALELRGVELEKRLRAAEG-------------------------------
MICAL-L2_Xenopus  784  ----------ARPEYIPEEEIQRQLQIIEQELDALEQQGVEMEKQLRICDG-------------------------------
                                         : :  . : :      **  *  :*  :*

                   ---------------------------H2-----------------------
                    .    d---a--d---a--d---a--d-d-a-ad-d---a--d---a--d
MICAL1_Human      PEQQKKLWVGQLLQLVDKKNSLVAEEAELMITVQELNLEEKQWQLDQELRGYMNREENL 1021
MICAL1_Mouse      PEQQKKLWLDQLLRLIQKKNSLVTEEAELMITVQELDLEEKQRQLDHELRGYMNREETM
MICAL1_Chicken    PADQKTQWMNQLLYLVQKKNSLMVEESDLMITVQELKLEEQQWQLDQELRWYIETEEAL
MICAL1_Zebrafish  GD---PEIIDQWIELVQEKNNLLSEESDLMVASRQLELEDKQSMLEMELRRRYMEMDDSE
MICAL_FruitFly    TKDNDEKLLKELLEIWRNITALKKRDEELTIRQQELQLEYRHAQLKEELNLRLSCNKLD
MICAL_ApisFlorea  SDREEADLLREWFDLMKERTELRRYEKELLVRAQEVQLEDRHERLQQELRERLADDDNK
MICAL3_Human      GKKDDPKLMQEWFKLVQEKNAMVRYESELMIFARELELEDRQSRLQQELRERMAVEDHL
MICAL3_Mouse      GKKDDPKLMQEWFKLVQEKNAMVRYESELMIFARELELEDRQSRLQQELRERMAVEDHL
MICAL3_Chicken    GKKDDPKLMQEWFKLVQEKNALVRYESELMIFARELELEDRQSRLQQELRERMAVEDHL
MICAL3a_Zebrafish GKKDDPSLMHQWFKLVQEKNALVRYESELMIFARELELEDRQSRLQQELRERMAVDDHL
MICAL3b_Zebrafish GKKDDPKLMQEWFKLVQEKNALVRYESELMIFARELELEDRQSRLQQELRERMAIDDHL
MICAL3_Lizard     GKKDDPKLMQEWFKLVQEKNALVRYESELMIFARELELEDRQSRLQQELRERMAVEDHL
MICAL-L1_Human    -EGREDDMLVDWFKLIHEKHLLVRRESELIYVFKQQNLEQRQADVEYELRCLLNKPEKD
MICAL-L1_Mouse    -EGSEDDMLVDWFKLIHEKHLLVRRESELIYVFKQQNLEQRQADVEFELRCLLNKPEKD
MICAL-L1_Chicken  -DSPEDSLLVDWFKLIHEKHMLVRHESELIYIFKQQNLEQRQADVEYELRCLLNKPEKD
MICAL-L_FruitFly  NSKEVEDLILQLFELVNEKNELFRRQAELMYLRRQHRLEQEQADIEHEIRVLMGQPEHN
MICAL-L_Lizard    MEAPEDGLLVDWFKLIHEKHMLVRRESELIYIFKQQNLEQRQADVEFELRCLLNKPEKE
MICAL-L2_Human    -DDAEDSLMVDWFWLIHEKQLLLRQESELMYKSKAQRLEEQQLDIEGELRRLMAKPEAL
MICAL-L2_Mouse    -DASEDSLMVDWFRLIHEKQLLLRLESELMYKSKDQRLEEQQLDLQGELRRLMDKPEGL
MICAL-L2_Xenopus  -DESEDTLMVDWFKLIHEKQLLLRQESELNYISKQQALEDKQSNIETELRNLMKKADHL
                          : : : :  :   :   : :*    :   ** .:  :. *:.  :   .

                     ------------------------H3---------
                             d---a--d---a--d---a
MICAL1_Human      KTAADRQAEDQVLRKLVDLVNQRDALIRFQEERRLSELALGTGAQG---- 1067
MICAL1_Mouse      KTEADLQSENQVLRKLLEVVNQRDALIQFQEERRLREMPA---------- 1048
MICAL1_Chicken    KTPEDRAAEQQILAQLLKVVDKRNALIHMQEEKRLSELHP---------- 1142
MICAL1_Zebrafish  KSPEQQKHEAEILQEMLDVVDMRDSLVAFLEEKRLKEVNDQFNSSL-15- 1214
MICAL_FruitFly    KSSADVAAEGAILNEMLEIVAKRAALRPTASQLDLTAAGSASTSAE-18- 4720
MICAL_ApisFlorea  KTSADVKKEGEILTEMLEIVAKRDSLIALLEEERQRYQDEDRDLEA-21- 2672
MICAL3_Human      KTEEELSEEKQILNEMLEVVEQRDSLVALLEEQRLREREEDKDLEA-12- 2002
MICAL3_Mouse      KTEGELSEEKKILNEMLEVVEQRDSLVALLEEQRLREKEEDKDLEA-12- 1993
MICAL3_Chicken    KTDEELSEEKRILNEMLEVVEQRDSLVALLEEQRLREKEEDKDLEA-12- 2186
MICAL3a_Zebrafish KGEEELAEERRILSEMLDVVEQRDALVALLEEQRVREKEEDSDLEA-12- 1673
MICAL3b_Zebrafish KTEEELAEEKQILNEMLEVVEQRDSLVALLEEQRLREKEEDKDLEA-12- 1994
MICAL3_Lizard     KTDDELSEEKRILNEMLEVVEQRDSLVALLEEQRLREKEEDKDLEA-12- 2255
MICAL-L1_Human    WTEEDRAREKVLMQELVTLIEQRNAIINCLDEDRQREEEEDKMLEA-45- 863
MICAL-L1_Mouse    WTDEDRAREKVLMQELMTLIEQRDAIVNCLDEDRQREEEEDKMLET-45- 870
MICAL-L1_Chicken  WTEEDRVREKVLMQELVTIIEQRNAIVNCLDEDRQREEEEDKMLEA-45- 953
MICAL-L_FruitFly  KTDSDKAHEEVLINRLVKVVEMRNEVIDSLETDRVREAREDMSIKN-71- 1010
MICAL-L_Lizard    WTDDDRVREKVLMQELVTIIEQRNAIVNCLDEDRQREEEEDKMLEA-45- 952
MICAL-L2_Human    KSLQERRREQELLEQYVSTVNDRSDIVDSLDEDRLREQEEDQMLRD-30- 904
MICAL-L2_Mouse    KSPQDRQREQELLSQYVNTVNDRSDIVDFLDEDRLREQEEDQMLEN-29- 1009
MICAL-L2_Xenopus  KTPGESEREKELLDQLLLIVNDRSEIVDCLDEDRIREKEEDEMMNA-39- 967
                      :   *  :: . :  :  *  :    .
```

Supplementary Figure 4: MICAL C-terminal domain fold conservation

Alignment of C-terminal regions of selected MICAL sequences. The output reflects the degree of sequence similarity among the proteins. Orange letters: conserved residues implicated in interaction between Helix-1 and Helix-2 and red letters: residues implicated in interaction between Helix-2 and Helix-3. Glycine and proline residues frequently found in loop structures of proteins are shown in brown. Residues matching the consensus pattern of heptad repeat motif for canonical coiled-coils are annotated above the sequence for MICAL1 with *a* and *d* letters. Residues mutated to define the surface S1 and S2 are indicated with highlights using a color code as defined in Fig.5a (Surface 1 in yellow (S1: E946, V950, E953, V971, L975, V978) and Surface 2 in orange (S2: R1012, M1015, L1034, V1038). The side chains of aa I1048 and R1055 essential for Rab35 interaction are displayed in white with highlighted red contour). Conserved residues on the surface of Helix-2 and Helix-3 (as defined in Fig. 5b) are indicated with green triangles below the sequences.

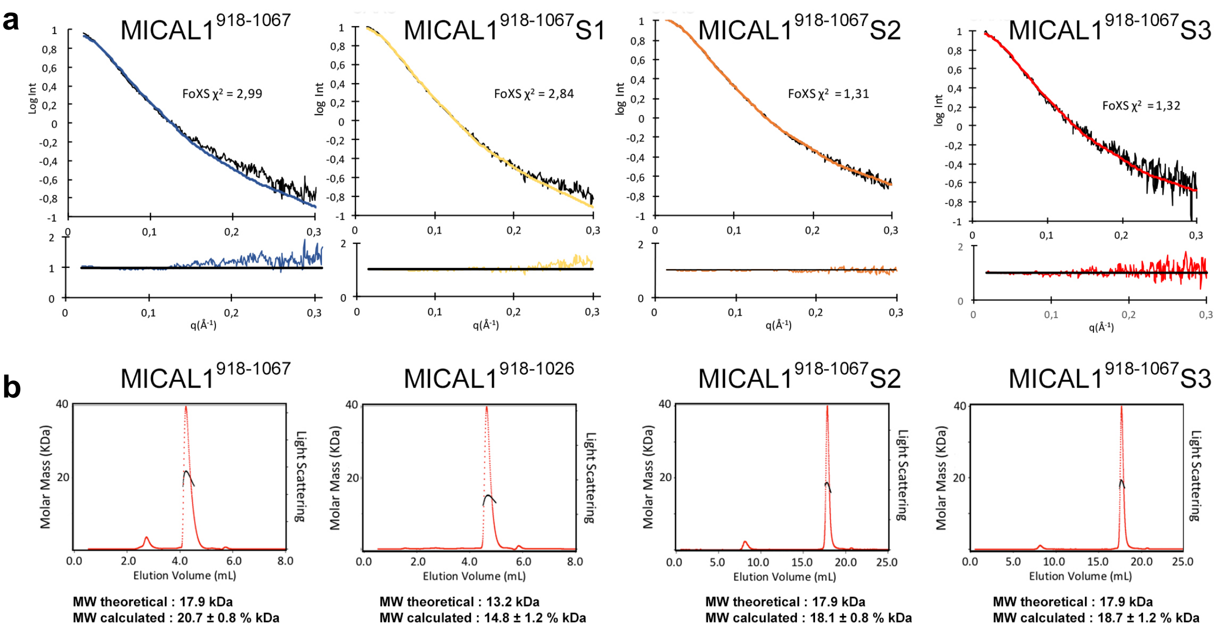

Supplementary Figure 5: SAXS and MALS analysis of MICAL1 constructs

(a) Comparison of SAXS profiles calculated from atomic model (colored lines) against SAXS experimental data (black curve). The residual plots are for q($Å^{-1}$) (x axis) versus experimental intensity divided by model calculated intensity (y axis). The SAXS data of the S1 mutant (E946K, V950D, E953K, V971E, L975R, V978E), S2 mutant (R1012E, M1015R, L1034K, V1038E) and S3 mutant (E1001R, V1038E, V1041E, I1048R, R1055E) are consistent with a conserved fold despite the introduction of mutations.

(b) SEC-MALS elution profiles displaying the plots of the UV signal (red line) at 280 nm and plot of molar mass (black dashed line) vs. elution volume. Molar masses determined by MALS analysis (dotted lines) across the peaks of eluted protein from a Superdex 200 (for MICAL1$^{918\text{-}1067}$-S2 and MICAL1$^{918\text{-}1067}$-S3) size exclusion column or X-Bridge BEH SEC 200Å (for MICAL1$^{918\text{-}1067}$ and MICAL1$^{918\text{-}1026}$) column. All the proteins are eluted as single molecular species.

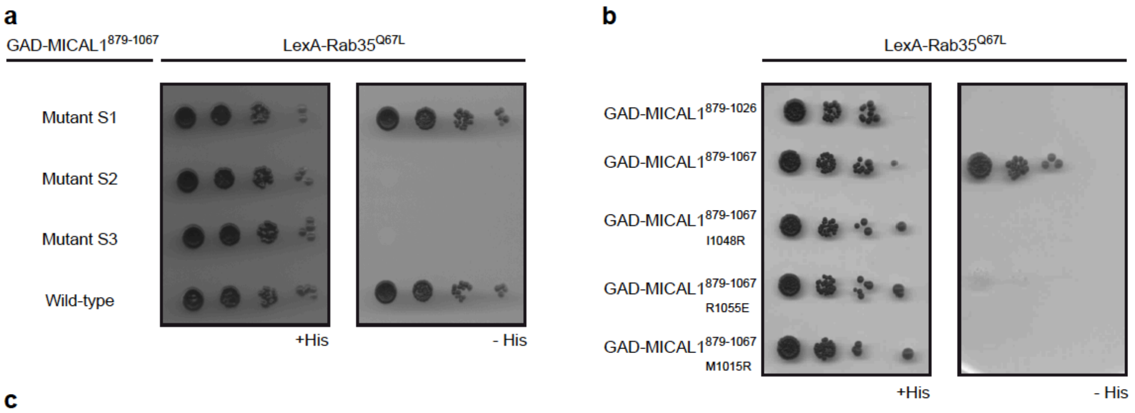

c

GAD / LexA	MICAL1 879-1067	MICAL1 879-1026 (Δ41)	MICAL1$^{879-1067}$									
			Mutant S1	Mutant S2	R1012E M1015R	R1012E	M1015R	Mutant S3	E1001R	E1001R I1048R R1055E	I1048R	R1055E
Rab35^{Q67L}	+	-	+	-	-	+/-	-	-	+	-	-	-

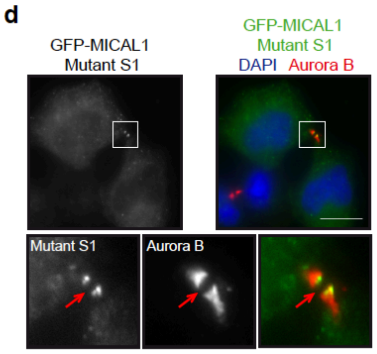

Supplementary Figure 6: Interactions between Rab35 and WT or mutant MICAL1

(a-b) *S. cerevisiae* reporter strain expressing indicated GAD- and LEX fusion proteins were grown on selective medium with or without Histidine.

(c) Summary of all the interaction between Rab35^{GTP} and MICAL1 mutants tested by yeast-two hybrids.

(d) HeLa cells transfected with GFP-MICAL1 Mutant S1 (green) were stained with Aurora B (red) and DAPI (blue). Scale bar, 10 μm.

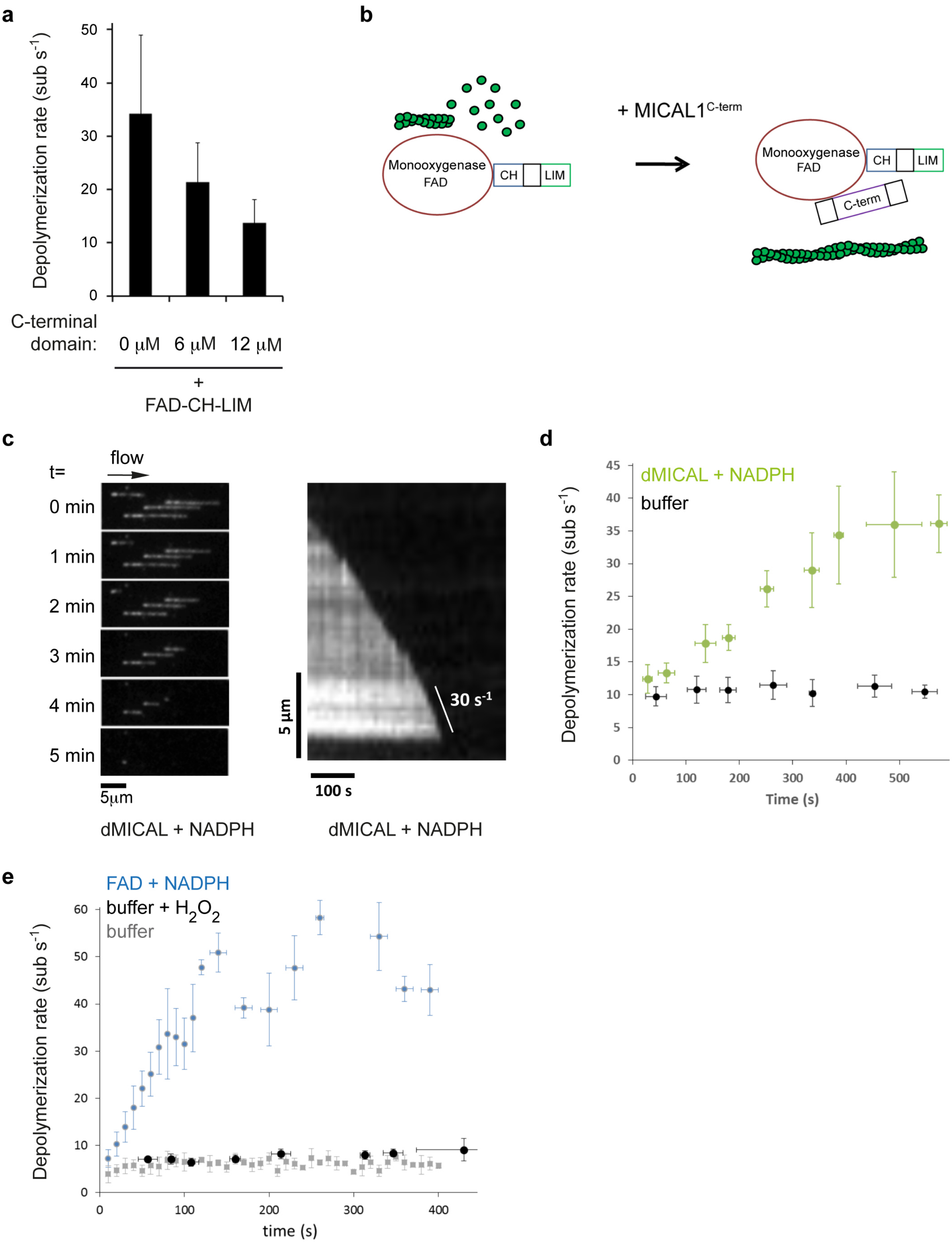
a
Depolymerization rate (sub s-1)
C-terminal domain:
0 µM
6 µM
12 µM
+
FAD-CH-LIM
b
Monooxygenase FAD
CH
LIM
+ MICAL1C-term
C-term
c
flow
t=
0 min
1 min
2 min
3 min
4 min
5 min
5µm
dMICAL + NADPH
5 µm
30 s-1
100 s
dMICAL + NADPH
d
dMICAL + NADPH
buffer
Depolymerization rate (sub s-1)
Time (s)
e
FAD + NADPH
buffer + H2O2
buffer
Depolymerization rate (sub s-1)
time (s)

Supplementary Figure 7: *Drosophila* MICAL has the same effects as human MICAL1 on actin filaments

(a) Depolymerization rates measured on ADP-actin filaments anchored on to the surface by inactivated myosins (as in Fig. 6d and f). Depolymerizing filaments are exposed to 120 µM NADPH, with 600 nM human FAD-CH-LIM and (from left to right) 0, 6 or 12 µM human MICAL1-C-terminal domain. 10-13 filaments quantified per condition. Error bars represent standard deviations.

(b) Model for inactivation of the redox enzyme MICAL1 by its C-terminal domain. Actin is in green.

(c) Timelapse images showing a typical set of ADP-actin filaments exposed to 600 nM dMICAL FAD-CH + 120 µM NADPH, in our microfluidics setup. The flow runs from left to right, the filament pointed ends (left hand side) are anchored to the coverslip surface, and the barbed ends (right hand side) depolymerize freely (left). Kymograph of a depolymerizing filament from the same experiment (right).

(d) Average depolymerization rates over time, for ADP-actin filaments exposed to 600 nM dMICAL FAD-CH + 120 µM NADPH (green) or to buffer alone (black). Error bars are standard deviations (right). 19-25 filaments quantified per condition.

(e) Using our microfluidics setup we monitored the barbed end depolymerization of ADP-F-actin in the presence of 40 mM H_20_2 (black) and compare it to data from Fig. 6B for filaments depolymerizing in the presence of buffer (gray) and in the presence of 600 nM human FAD + 120 µM NADPH (blue). We also verified the potency of our H_20_2 (data not shown) by monitoring the elongation of filaments from 1µM G-actin incubated with 40 mM H_20_2 for 20 minutes at 20°C and found that, in ageement with previous reports[5], H_2O_2 oxidizes G-actin and hinders polymerization: filaments elongated from H_20_2-treated monomers at 2.4 ± 0.3 sub/s (StdDev, N=12), compared to 7.8 ± 0.8 sub/s (StdDev, N=12) in control experiments.

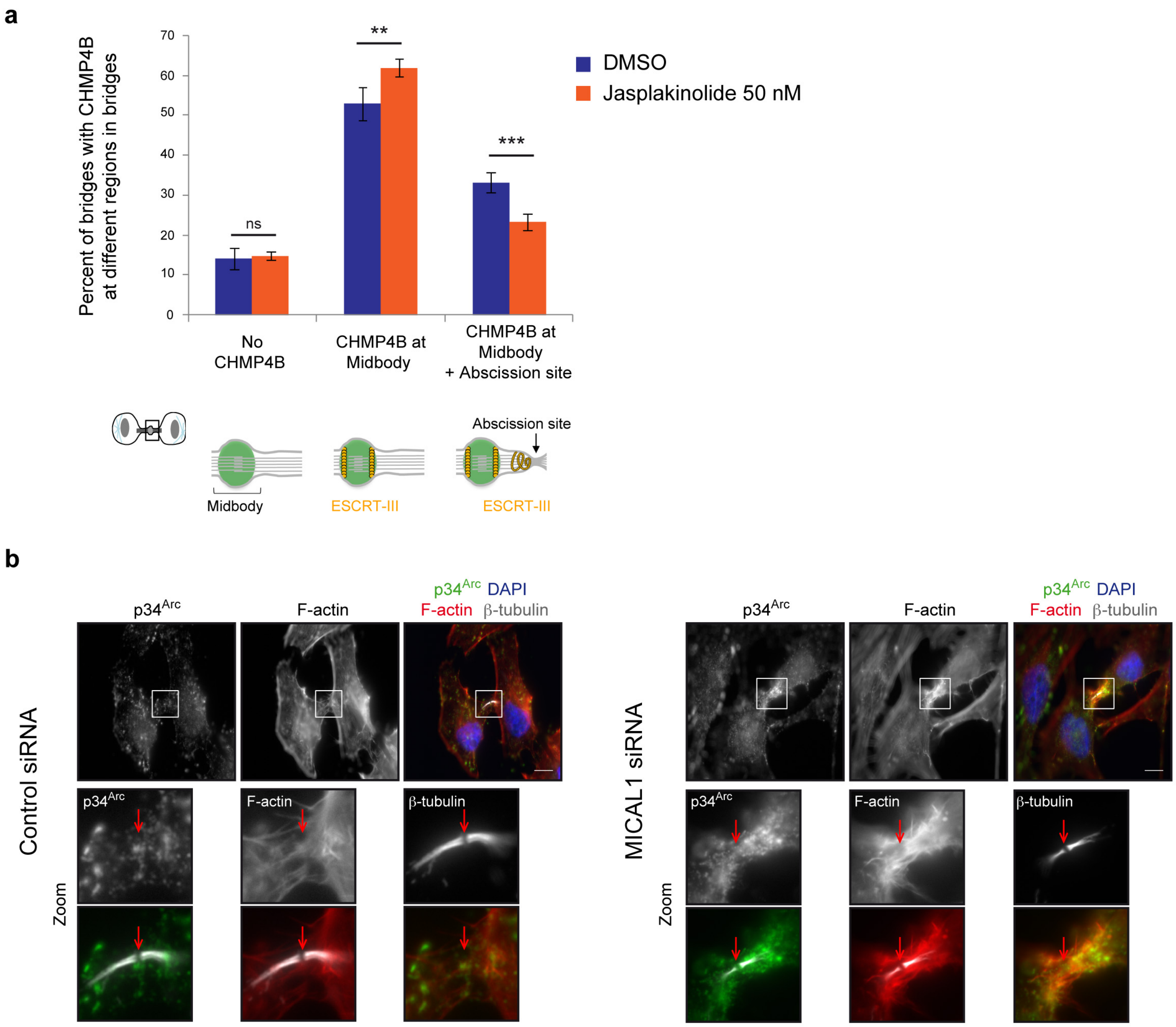

Supplementary Figure 8: F-actin stabilization impairs the recruitment of CHMP4B at the abscission site. MICAL1 depletion induces accumulation of Arp2/3 complex protein p34^{Arc} at the bridge

(a) Percentage of bridges with no CHMP4B, with CHMP4B only at the midbody, and with CHMP4B at the midbody + at the abscission site in cells treated with either DMSO or Jasplakinolide (N=4). Error bars represent standard deviations. **: $p< 0.01$; ***: $p< 0.001$ (two-way ANOVA). n= 330-347 cells per condition.

(b) Staining of p34^{Arc} (green), F-actin (phalloidin, red), β-tubulin (gray) and DAPI (blue) in control- or MICAL1-depleted cells. Scale bars, 10μm.

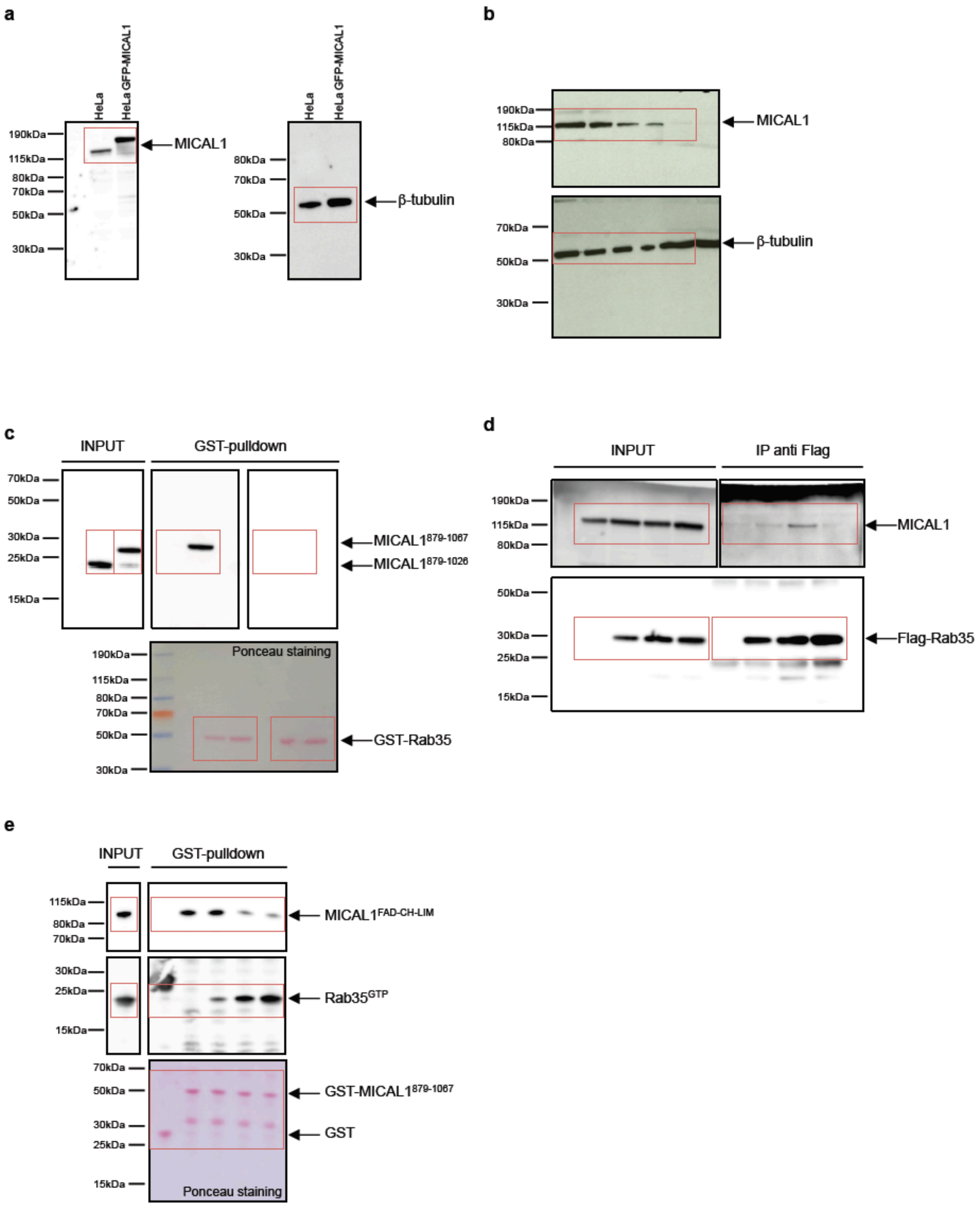

Supplementary Figure 9: uncropped images for presented Western Blots

(a) corresponds to Supplementary Fig. 1b; (b) corresponds to Fig. 2a; (c) corresponds to Fig. 4c; (d) corresponds to Fig. 4d; (e) corresponds to Fig. 6e.

C-term MICAL1		Rab35	Kd1	Kd2	mean Kd	N
MICAL1$^{918\text{-}1067}$		GDP-Rab35$_{1\text{-}175}$	n.b.*	n.b.*	-	-
MICAL1$^{918\text{-}1067}$		GppNHp-Rab35$_{1\text{-}175}$	8,3	4,5	6.4	1.09
MICAL1$^{918\text{-}1067}$		GppNHp-Rab35$_{1\text{-}199}$	4,5	5	4.8	1.07
MICAL1$^{879\text{-}1067}$		GppNHp-Rab35$_{1\text{-}175}$	13	13	13	0.8
MICAL1$^{918\text{-}1026}$		GppNHp-Rab35$_{1\text{-}175}$	n.b.*	n.b.*	-	-
MICAL1$^{918\text{-}1067}$	S1[a]	GppNHp-Rab35$_{1\text{-}175}$	7,9	6,4	7.2	0.95
MICAL1$^{918\text{-}1067}$	S2[b]	GppNHp-Rab35$_{1\text{-}175}$	n.b.*	n.b.*	-	-
MICAL1$^{918\text{-}1067}$	I1048R	GppNHp-Rab35$_{1\text{-}175}$	n.b.*	n.b.*	-	-
MICAL1$^{918\text{-}1067}$	R1055E	GppNHp-Rab35$_{1\text{-}175}$	w.b.[+]	w.b.[+]	-	-
MICAL1$^{918\text{-}1067}$	M1015R	GppNHp-Rab35$_{1\text{-}175}$	w.b.[+]	w.b.[+]	-	-

Equilibrium dissociation constants (K_d) and stoichiometry constant (N) values were determined by ITC measurements.
[*] no binding
[+] very weak binding, fitting is not reliable
[a] S1 mutant (E946K, V950D, E953K, V971E, L975R, V978E).
[b] S2 mutant (R1012E, M1015R, L1034K, V1038E).

Supplementary Table 1: Isothermal Titration Calorimetry measurements for Rab35 and C-terminal MICAL1 constructs

	Peak SAD SeMet
**Data collection**	
Space group	P $4_3 22$
Cell dimensions	
a, *b*, *c* (Å)	52.78, 52.78, 157
α, β, γ (°)	90, 90, 90
Wavelength	0.978870
Resolution (Å)[a]	43.8 - 3.3 (3.49 - 3.3)
R_{sym}	3.7 (32.1)
R_{meas}	4 (34.8)
$I/\sigma(I)$	26.56 (4.67)
$CC_{1/2}$	100 (98.4)
Completeness (%)	99.6 (97.9)
Redundancy	7 (6.6)
**Refinement**	
Resolution (Å)	37.32 - 3.3 (3.418 - 3.3)
No. reflections	3730 (356)
R_{work}/R_{free}	0.2715 (0.3131)/0.3068 (0.4240)
No. atoms protein	956
B factors protein	120.59
R.m.s deviations	
Bond lengths (Å)	0.005
Bond angles (°)	0.62
Favoured/allowed/outlier Ramachandran angles	96%/4.3%/0%

[a] Values in parentheses are for highest-resolution shell.
The crystallized MICAL1 fragment corresponds to residue range: 918-1067. The loops: 918-924, 955-963, 1017-1019 and 1060-1067 - are missing in the structure.

Supplementary Table 2: Data collection and refinement statistics for the MICAL1 C-terminal domain (PDB ID code 5IE0)

Supplementary References

1. Knight, P.J. *et al.* The predicted coiled-coil domain of myosin 10 forms a novel elongated domain that lengthens the head. *The Journal of biological chemistry* **280**, 34702-34708 (2005).
2. Sievers, F. *et al.* Fast, scalable generation of high-quality protein multiple sequence alignments using Clustal Omega. *Molecular systems biology* **7**, 539 (2011).
3. Senkovich, O. *et al.* Structure of a complex of human lactoferrin N-lobe with pneumococcal surface protein a provides insight into microbial defense mechanism. *Journal of molecular biology* **370**, 701-713 (2007).
4. Barta, M.L. *et al.* The structures of coiled-coil domains from type III secretion system translocators reveal homology to pore-forming toxins. *Journal of molecular biology* **417**, 395-405 (2012).
5. DalleDonne, A. & Milzani, R. H2O2-treated actin: assembly and polymer interactions with cross-linking proteins. *Biophysical journal* **69**, 2710-2719 (1995).
